# Most canine ameloblastomas harbor HRAS mutations, providing a novel large-animal model of RAS-driven cancer

**DOI:** 10.1038/s41389-019-0119-1

**Published:** 2019-02-11

**Authors:** Persiana S. Saffari, Natalia Vapniarsky, Anna S. Pollack, Xue Gong, Sujay Vennam, Andrew J. Pollack, Frank J. M. Verstraete, Robert B. West, Boaz Arzi, Jonathan R. Pollack

**Affiliations:** 1 Department of Pathology, Stanford University School of Medicine, Stanford, CA USA; 2 Department of Pathology, Microbiology & Immunology, UC Davis School of Veterinary Medicine, Davis, CA USA; 3 Department of Surgical & Radiological Sciences, UC Davis School of Veterinary Medicine, Davis, CA USA

## Abstract

Canine acanthomatous ameloblastomas (CAA), analogs of human ameloblastoma, are oral tumors of odontogenic origin for which the genetic drivers have remained undefined. By whole-exome sequencing, we have now discovered recurrent *HRAS* and *BRAF* activating mutations, respectively, in 63% and 8% of CAA. Notably, cell lines derived from CAA with HRAS mutation exhibit marked sensitivity to MAP kinase (MAPK) pathway inhibitors, which constrain cell proliferation and drive ameloblast differentiation. Our findings newly identify a large-animal spontaneous cancer model to study the progression and treatment of RAS-driven cancer. More broadly, our study highlights the translational potential of canine cancer genome sequencing to benefit both humans and their companion animals.

## Introduction

As do humans, domestic dogs develop spontaneous cancers with genetic and environmental influences^1,2^. Common cancers in dogs include lymphoma, osteosarcoma, mammary carcinoma, hemangiosarcoma, oral melanoma, and mast cell tumors, among others. Canine cancers display strong similarities to their human counterparts in histopathology, tumor genetics, and clinical behavior. With millions of pet dogs cared for into old age (and about half developing cancer), dogs offer a largely untapped resource for new cancer insight, as well as advantageous models for preclinical testing^3^. Toward this end, and enabled by the completion of the canine reference genome^4^, incipient efforts are underway to systematically sequence canine cancer genomes^5–7^.

Canine acanthomatous ameloblastomas (CAAs) are odontogenic tumors of the jaw, thought to represent the counterpart of human ameloblastoma (acanthomatous histologic variant)^8^. CAAs share with human ameloblastoma their histology, propensity to infiltrate bone while rarely metastasizing, and presumptive origin from the ameloblast (enamel secreting) cell lineage^9^, though non-odontogenic origins have also been speculated. CAAs are found across diverse dog breeds and notably occur far more commonly than do human ameloblastomas^10^. Current recommended treatment of CAA is surgical excision. While human ameloblastomas harbor driver mutations in the mitogen-activated protein kinase (MAPK) pathway (including *BRAF*, *KRAS*, *NRAS*, *HRAS* and *FGFR4*) and Hedgehog pathway (*SMO*)^11,12^, the drivers of CAA have not been known.

## Results

### Frequent HRAS mutations in CAA

**Fig. 1:**
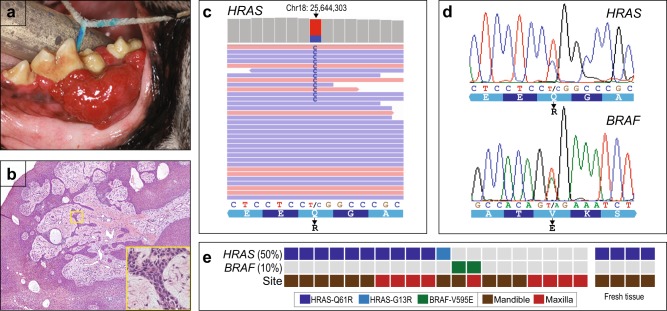
Whole-exome sequencing (WES) of canine acanthomatous ameloblastoma (CAA) identifies recurrent *HRAS* and *BRAF* mutations. **a** Mandibular CAA case prior to resection. **b** Histologic architecture (hematoxylin–eosin (H&E) stain) of typical CAA case; note tumor epithelium (violet) interdigitates with stroma (pink). Inset shows tumor region at higher magnification. CAA formalin-fixed paraffin-embedded (FFPE) tissue blocks (dated 2007–2015) were retrieved from the clinical archives of the Department of Pathology, UC Davis School of Veterinary Medicine, and H&E-stained sections reviewed by a trained veterinary pathologist (N.V.). **c** Integrated Genome Viewer display of mapped reads from WES of CAA case harboring HRAS-Q61R mutation. Red and blue reads map to plus and minus strands, respectively; only a subset of mapped reads is shown. WES was done on 16 CAA samples; while this was an exploratory study, sample sizes of 10–15 should provide 80% power to identify driver mutations if present at ≥20–30% frequency. Genomic DNA was extracted from CAA FFPE tissue scrolls using the Qiagen (Germantown, MD, USA) DNA FFPE Tissue Kit. WES was done using the Agilent (Santa Clara, CA, USA) SureSelect Canine All Exon Kit, following modifications recommended for FFPE-derived DNA samples. Barcoded WES libraries were sequenced (101 bp × 2) on an Illumina HiSeq2500 or 4000 instrument (Stanford Genome Sequencing Service Center) to an average 116× mean base pair coverage. Raw reads were aligned to the dog genome (CanFam3.1) using BWA^21^. Single-nucleotide variants (SNVs) were called using SAMtools^22^ mpileup and, in the absence of matched normal, restricted to 597 canine gene orthologs of known human cancer genes (the union of Cancer Gene Census and FoundationOne gene lists) (Table S2). SNVs were annotated using the Ensembl Variant Effect Predictor^23^. Subsequently, SNVs were filtered to exclude known germline variants (SNPs) and to retain only those SNVs with High evidence (read depth ≥20; minor allele frequency 20–50%) and High consequence (missense, stop-gain, or splice donor/acceptor variants), yielding 171 SNVs (in 91 genes) across 16 tumors (Table S4). To further distinguish likely somatically acquired SNVs from personal germline SNPs, we focused only on those SNVs occurring at the orthologous position of known human cancer hotspot mutations^24^ (Table S3), determined from the Catalogue of Somatic Mutations in Cancer (COSMIC)^25^. Finally, we performed manual inspection of reads spanning HRAS-61, HRAS-13, and BRAF-595, identifying one additional HRAS-Q61R case (CAA-20) with mutant allele frequency 11%, missed by the automated SNV caller. All WES data are available from NCBI SRA (accession PRJNA516699). **d** Sanger sequencing validation of HRAS-Q61R and BRAF-V595E mutations in two different CAA cases. All *HRAS* and *BRAF* mutations identified by WES were confirmed by PCR amplification followed by Sanger sequencing. The PCR/sequencing primers used are available in Table S7. **e** Summary of *HRAS* and *BRAF* mutations across the 20 CAA FFPE and 4 fresh tissue cases surveyed; anatomic site indicated (see color key). Note, no *HRAS* or *BRAF* mutations were identified outside of the mutation hotspots in any of the samples

To identify cancer-driving mutations in CAA, we carried out whole-exome sequencing (WES) of formalin-fixed paraffin-embedded (FFPE) tumor tissue from 16 prototypical CAA cases from diverse breeds (Fig. 1a, b and Tables 1, S1). We then used PCR/Sanger sequencing to confirm select mutations in the discovery set plus additional specimens (together totaling 20 CAA cases). Because we lacked matched normal tissue (useful to exclude personal germline single-nucleotide variants (SNVs)), our analysis focused on the canine orthologs of ~600 known human cancer genes and, within that set, known mutation “hotspot” sites (Fig. S1 and Tables S2, S3).

**Table 1:** Canine acanthomatous ameloblastoma case characteristics

	Case ID	Location	Breed	Age (years)	Sex	Weight (kg)	Mutation	Read depth^a^	VAF
FFPE tissue cases	CAA-01	Mandible	German Shepherd	8	MC	42			
	CAA-02	Mandible	Labrador Retriever	13	FS	31			
	CAA-03	Mandible	Labrador Retriever	11	FS	33	HRAS-Q61R	72	0.278
	CAA-04	Mandible	Pit Bull Terrier	10	FS	27	HRAS-Q61R		
	CAA-05	Mandible	Shetland Sheepdog	11	MC	22	BRAF-V595E	197	0.289
	CAA-06	Mandible	Border Collie	8	MC	29	HRAS-Q61R	63	0.270
	CAA-07	Mandible	Australian Shepherd	9	MC	31	HRAS-G13R	38	0.421
	CAA-08	Mandible	Basset Hound	15	MC	36	HRAS-Q61R		
	CAA-09	Mandible	Cocker Spaniel	9	FS	15	HRAS-Q61R	95	0.263
	CAA-10	Mandible	Husky mix	10	FS	39			
	CAA-11	Mandible	Chesapeake Bay Retriever	6	MC	33	HRAS-Q61R		
	CAA-12	Maxilla	Samoyed	10	FS	36	HRAS-Q61R	63	0.238
	CAA-13	Maxilla	Beagle	10	MC	12			
	CAA-14	Maxilla	Collie	11	MC	30			
	CAA-15	Maxilla	Labrador Retriever	12	MC	39	BRAF-V595E		
	CAA-16	Maxilla	Collie	11	MC	31			
	CAA-17	Maxilla	Standard Poodle	7	MC	25			
	CAA-18	Maxilla	Labrador Retriever	11	FS	26	HRAS-Q61R	90	0.267
	CAA-19	Maxilla	English Bulldog	10	MC	NA	HRAS-Q61R	57	0.456
	CAA-20	Maxilla	Beagle mix	5	MC	17	HRAS-Q61R	101	0.109
Fresh tissue cases	CAA-21	Mandible	Labrador Retriever	8	FS	31	HRAS-Q61R		
	CAA-22	Mandible	Terrier mix	8	FS	22	HRAS-Q61R		
	CAA-23	Mandible	Great Dane	9	FS	53	HRAS-Q61R		
	CAA-24	Mandible	Standard Poodle	3	MC	40	HRAS-Q61R		

*MC* male castrated, *FFPE* formalin-fixed paraffin-embedded, *FS* female spayed, *VAF* variant allele frequency

^a^Read depth at mutated base

Strikingly, 11 of the 20 (55%) CAA cases carried activating *HRAS* mutations (10 HRAS-Q61R and 1 HRAS-G13R), and 2 of the 20 (10%) carried activating *BRAF* mutations (BRAF-V595E, orthologous to the human BRAF-V600E driver mutation) (Fig. 1c–e and Tables 1, S4). In the seven remaining CAA cases, no driver hotspot mutation was identified. *HRAS* and *BRAF* mutation allele frequencies (range 11–46%; mean 29%) were consistent with somatically acquired mutations (i.e., admixed with normal stroma), which we confirmed in three CAA cases by laser microdissection (and PCR/Sanger sequencing) of separate tumor epithelium and stroma (Fig. S2). In distinction from human ameloblastomas, where *BRAF* and *SMO* mutations are preferentially localized, respectively, to mandibular and maxillary tumors^12^, the canine *HRAS* and *BRAF* mutations occurred in both anatomic sites (Fig. 1e), and no canine *SMO* mutations were identified. We also used the WES reads to infer DNA copy number alterations (CNAs); all but one CAA case exhibited relatively flat CNA profiles (Fig. S3).

### HRAS mutations confer sensitivity to MAPK pathway inhibition

**Fig. 2:**
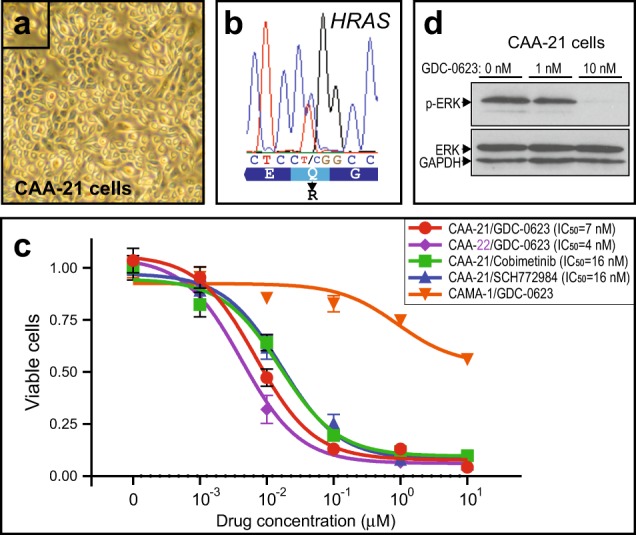
Canine acanthomatous ameloblastoma (CAA)-derived cell lines harbor *HRAS* mutation and are sensitive to select mitogen-activated protein kinase (MAPK) pathway inhibitors. **a** CAA cell line (CAA-21) generated by conditional reprogramming. Fresh CAA tissue (dated 2017–2018) was obtained from tumors excised as part of standard surgical treatment; use of surplus tumor tissue was exempt from IACUC approval. CAA cell lines (Table S1) were established by conditional reprogramming following published methods^26^. Briefly, fresh tumor tissue was minced, and then cells were disaggregated by Collagenase/Hyaluronidase using StemCell Technologies (Vancouver, BC, Canada) reagents and protocols, followed by Trypsin and Dispase. Cells were then filtered through a 40-µM cell strainer and plated in Complete F medium (conditioned by irradiated J2 strain mouse Swiss-3T3 fibroblasts and containing 10 µM ROCK inhibitor (Y-27632)). Cells were passaged by trypsinization, with no appreciable change in growth properties over >20 passages. Cell lines are available from J.R.P. upon request. **b** CAA cells retain HRAS-Q61R mutation, verified by PCR/Sanger sequencing. **c** MAPK inhibitor dose–response (inhibition) curves depict sensitivity to select MAPK inhibitors. IC_50_ values are indicated. MAPK inhibitors were obtained from Selleckchem (Houston, TX, USA). Drug testing was performed in complete F media (including Y-27632). 50K cells were plated per 6-well plate well (in duplicate) and then challenged with a 10-fold drug dilution series (or vehicle alone) for 72 h, with daily media/drug replacement. Cell viability was then measured by flow cytometry (BD Accuri C6) or Countess automated cell counter (Thermo Fisher, Waltham, MA, USA). IC_50_ values were calculated from dose–response (inhibition) curves using GraphPad Prism. All cell culture experiments were repeated at least twice with comparable results. CAMA-1 cells were obtained directly from the ATCC (Manassas, VA, USA) and cultured as recommended. **d** Verification of mitogen-activated extracellular signal-regulated kinase (ERK) inhibitor (GDC-0623) on-target activity in CAA cells; western blot indicates IC_50_ (phospho-ERK levels) at 1–10 nM. Western blots were done using primary antibodies against phospho-Erk1/2 (clone D13.14.4E) and Erk1/2 (clone 3A7) (Cell Signaling Technology, Danvers, MA), with detection by chemiluminescence and quantification by ImageJ

To further investigate MAPK pathway-driven CAA, we generated immortalized cell lines from fresh tissue of four additional CAA cases, by conditional reprogramming (i.e., culturing cells with Rho-associated protein kinase (ROCK) inhibitor and irradiated fibroblast conditioned media)^13^. All four cell lines harbored the HRAS-Q61R activating mutation (Fig. 2a, b and Table 1). Testing two of the CAA (HRAS-Q61R) cell lines, both were highly sensitive (at low nanomolar concentrations) to mitogen-activated extracellular signal-regulated kinase (MEK) inhibition by GDC-0623, an allosteric MEK inhibitor that also blocks feedback-mediated RAF/MEK activation^14^ (Fig. 2c). Inhibition of canine MEK activity was confirmed by phospho-extracellular signal-regulated kinase (phospho-ERK) western blot (Fig. 2d). To exclude nonspecific cell toxicity of GDC-0623, we tested CAMA-1 breast cancer cells, which we found consistent with published reports^15^ to be insensitive to MEK inhibition (Fig. 2c). CAA (HRAS-Q61R) cells were also highly sensitive to the MEK inhibitor cobimetinib (GDC-0973), though it has been reported less effective against mutant-RAS than mutant-BRAF-driven tumor models^14^, as well as the ERK inhibitor SCH772984, reported effective against RAS-driven cancer models^16^ (Fig. 2c).

**Fig. 3:**
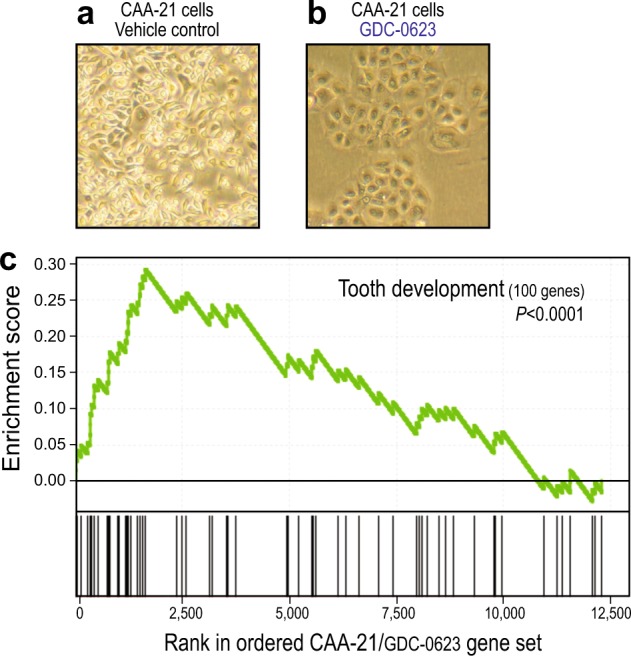
Transcriptome changes in canine acanthomatous ameloblastoma (CAA) cells induced by mitogen-activated extracellular signal-regulated kinase (MEK) inhibition. **a** Morphology of CAA cells treated with MEK inhibitor GDC-0623 (vs. vehicle control). **b** MEK inhibitor addition to CAA (HRAS-Q61R) cells generates a transcriptional response significantly enriched for tooth development genes. **c** Gene Set Enrichment Analysis (GSEA) enrichment score *P* value is indicated. Tooth development genes within the leading edge are listed in Table S5. For transcriptome sequencing, CAA-21 cells were plated in 6-well plate wells, and 1 µM GDC-0623 (or vehicle control) was added with daily media replacement for 72 h. RNA was isolated using the Qiagen RNeasy Kit, RNAseq libraries generated using Illumina (San Diego, CA, USA) TruSeq RNA Library Prep Kit v2, and barcoded RNAseq libraries sequenced (101 bp × 2) on an Illumina HiSeq2500 to an average depth of 20 million reads. Reads were mapped to the Ensembl-annotated (CanFam3.1) transcriptome using TopHat and Cufflinks^27^, and transcripts quantified as Reads Per Kilobase of transcript per Million mapped reads (RPKMs). Enrichment of tooth development genes^17^ was evaluated by GSEA^28^. All RNAseq data are available from NCBI SRA (accession PRJNA516699)

Interestingly, MEK inhibition not only blocked CAA (HRAS-Q61R) cell proliferation but also led to cell flattening reminiscent of cellular senescence and/or terminal differentiation (Fig. 3a, b). To further investigate, we profiled gene expression following MEK inhibition by GDC-0623 (vs. vehicle control). Notably, the genes upregulated by MEK inhibition were significantly enriched for tooth development genes^17^ (*P* < 0.0001; Gene Set Enrichment Analysis) (Fig. 3c and Table S5), supporting an odontogenic origin for CAA. Among these, the ameloblast-specific gene AMTN (Amelotin)^18^ was upregulated ~5000-fold (Table S6).

## Discussion

Here, by WES of CAA FFPE and subsequent fresh tissue specimens, we have in total identified *HRAS* activating mutations in 63% of cases (15 of 24) and *BRAF* activating mutations in 8% of cases (2 of 24). Together, over two thirds (71%) of CAA cases carry activating MAPK pathway mutations that should be targetable by existing Food and Drug Administration-approved or investigational drugs. Indeed, we demonstrate that CAA cells carrying HRAS-Q61R mutation are highly sensitive to MEK and ERK inhibition. Interestingly, MEK inhibition not only constrains cell proliferation but also appears to drive ameloblast differentiation, noted by the 5000-fold induction of the ameloblast-specific AMTN transcript.

While most CAA cases harbored *HRAS* or *BRAF* mutations, 29% (7 of 24) carried neither. Because we did not have matching normal DNA (helpful in distinguishing somatic mutations from personal germline variants), we limited our analysis to the canine orthologs of known human cancer gene hotspot mutations. Future studies that include matching normal DNA may reveal additional CAA-driver mutations, either within or outside the MAPK pathway, and should inform mutational burdens as well as signatures suggestive of particular mutational processes.

Additionally, while CAA cells with HRAS-Q61R showed sensitivity to MEK and ERK inhibitors, it remains to be determined whether single-agent therapies will be effective in vivo. For example, with human BRAF-mutant melanomas treated by BRAF inhibition, acquired resistance often develops, while dual BRAF and MEK inhibition has shown improved efficacy^19^.

Importantly, our findings newly identify a large-animal spontaneous tumor model of RAS/RAF-driven cancer, valuable for preclinical testing of MAPK pathway inhibitors. CAA could model MAPK pathway dependence, inhibitor sensitivity, and resistance not only for human ameloblastoma but potentially also for other RAS/RAF mutation-driven human cancers (e.g., thyroid cancer, lung cancer, pancreatic cancer, and melanoma). Surgical excision remains the mainstay treatment of human ameloblastoma, though targeted therapies (particularly MAPK pathway inhibitors) show promise^20^, and regimens might be optimized through preclinical testing in dogs. Our findings also offer more immediate translation in the management of CAA, for example, for compassionate use of MEK/ERK inhibitors in pet dogs that are not surgical candidates (e.g., because of tumor location, extent, or comorbidities). More broadly, our study demonstrates the feasibility, importance, and promise of dog genome sequencing and comparative oncogenomics studies and the commensal benefit to both humans and their companion animals.

## Supplementary information

Supplementary Figures

Supplementary Tables
