# Supplementary material for: Most canine ameloblastomas harbor HRAS mutations, providing a novel large-animal model of RAS-driven cancer
Source: Oncogenesis. 2019 Feb 11;8(2):11. doi: 10.1038/s41389-019-0119-1 (PMC6370874; doi:10.1038/s41389-019-0119-1)

Most canine ameloblastomas harbor HRAS mutations, providing a novel large-animal model of RAS-driven cancer

Persiana Saffari *et al*.

1. SUPPLEMENTARY TABLE TITLES

2. SUPPLEMENTARY FIGURES

Supplementary Tables

Provided as a single .xlsx file with multiple sheet tabs:

Table S1. Canine Acanthomatous Ameloblastoma (CAA) cases studied and results

Table S2. Orthologous cancer genes

Table S3. Mutation hotspots considered (shown only for SNV-bearing orthologous cancer genes meeting filtering criteria)

Table S4. Summary of identified SNVs meeting filtering criteria

Table S5. Leading edge tooth development genes (from GSEA)

Table S6. Genes upregulated in AAC-21 cells by GDC-0623 (top 50 genes shown)

Table S7. PCR/sequencing primers

Supplementary Figures

Fig. S1. SNV analysis pipeline. Illustrated are the sequential steps of the SNV analysis pipeline, along with identified SNV and gene numbers, and the corresponding Supplementary Tables. See Main text and Methods (Fig. 1c legend) for details.

Fig. S2. Sanger sequencing of laser-microdissected CAA tumor and stroma. (**a**) Images of laser capture microdissected CAA (case CAA-18) tumor epithelium (*above*) and stroma (*below*), showing the regions marked or excised from tissue (*left*), and retained on LCM cap (*right*). Laser capture microdissection (LCM) was performed on three CAA cases (two HRAS-Q61R, and one BRAF-V595E) using an Arcturus XT LCM system. DNA was isolated using PicoPure DNA Extraction Kit, and PCR done using 1ng input DNA and 40 cycles, followed by Sanger sequencing (Quintara Biosciences, Hayward, CA, USA). (**b**) Corresponding Sanger sequencing chromatograms of HRAS-61 from CAA tumor epithelium (*above*) and stroma (*below*). Note mixed peak (HRAS-Q61R) present only in microdissected tumor tissue.

Fig. S3. Copy number alteration profiles of CAA. Shown are $\log_2$ copy number values (with calculated segmental alteration values in orange), inferred by WES read counts, across the 38 canine autosomes and X chromosome. CNA profiles are shown for 3 representative cases: (**a**) The only one of 16 profiles exhibiting whole chromosome aneuploidies (here, gain of chromosomes 4, 13, 14, 23, 24 and 30); (**b**) Typical "flat" CNA profile (i.e. no aneuploidies) of

CAA from female dog; (**c**) Typical flat CNA profile of CAA from male dog (note apparent "loss" of X chromosome). CNA profiles were generated from WES data using CNVkit (Talevich et al., 2016).

Supplementary References

Talevich E, Shain AH, Botton T, Bastian BC (2016). CNVkit: Genome-Wide Copy Number Detection and Visualization from Targeted DNA Sequencing. *PLoS Comput Biol* **12:** e1004873.

Supplementary Figure 1

SNV analysis pipeline (16 CAA samples)

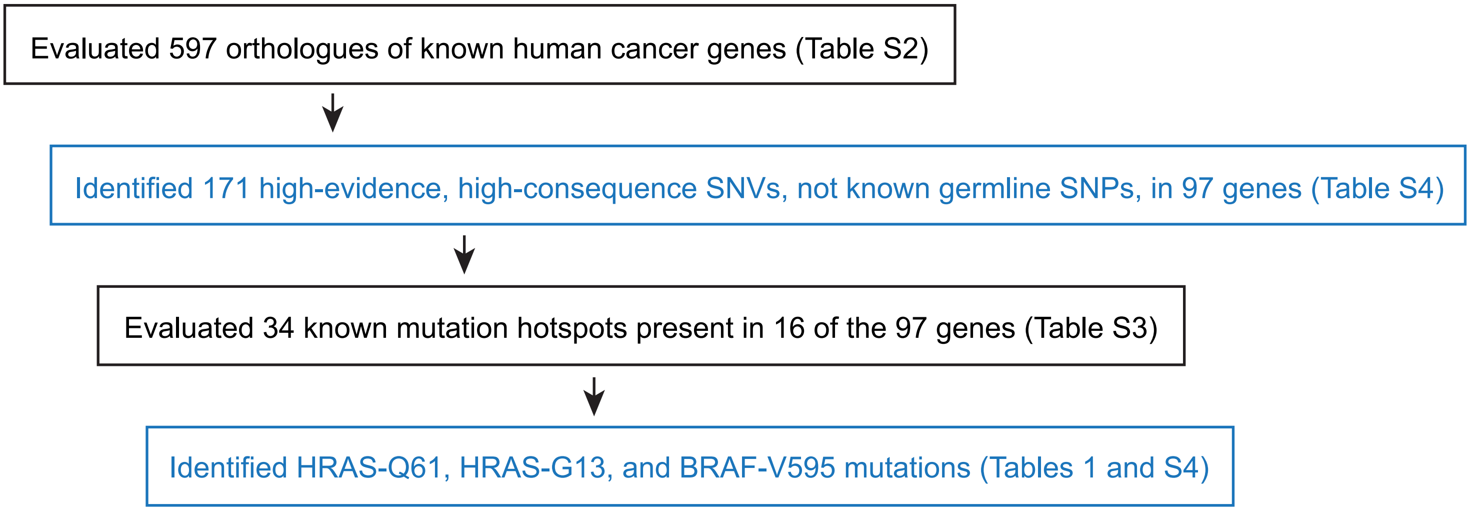

Supplementary Figure 2

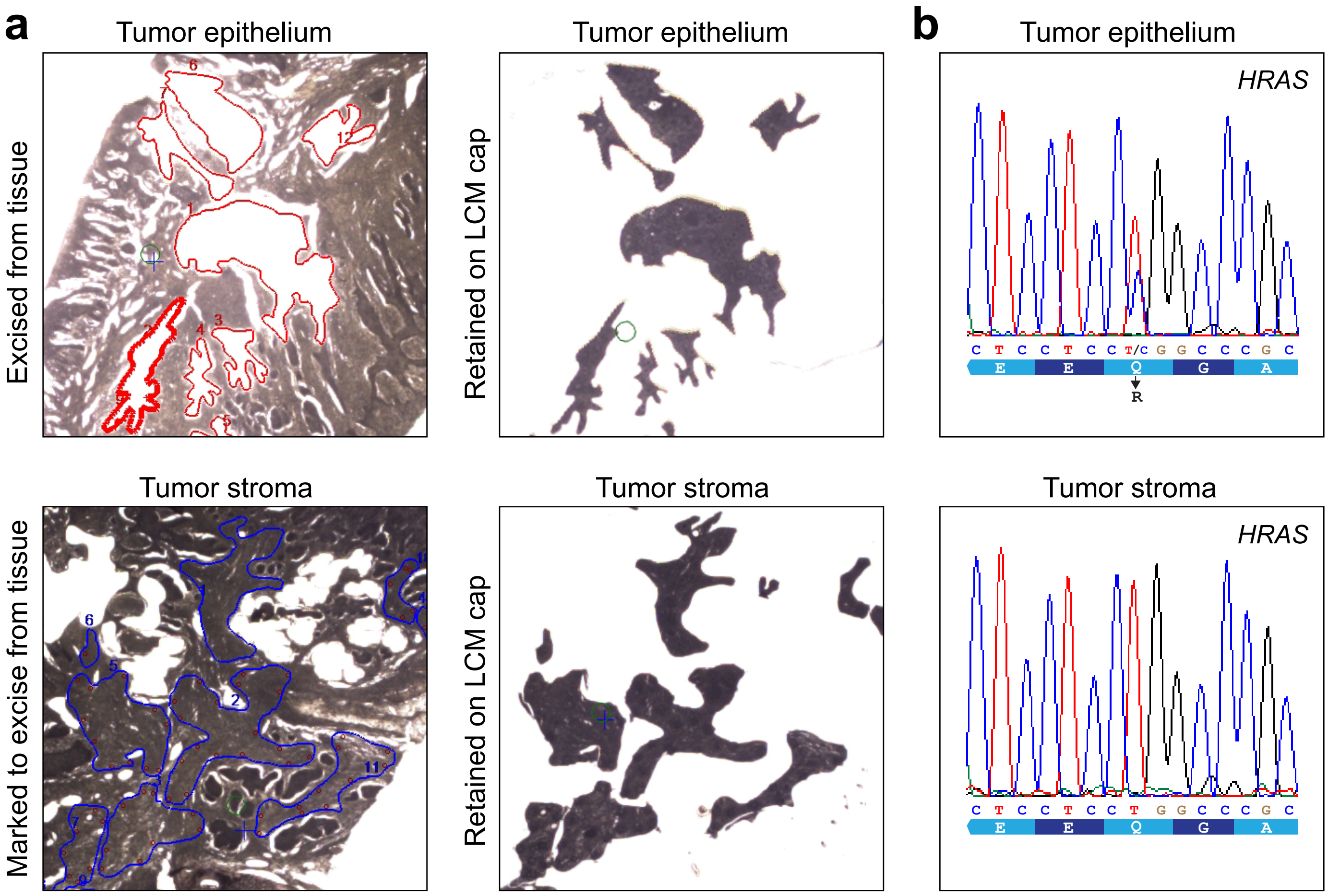

Supplementary Figure 3

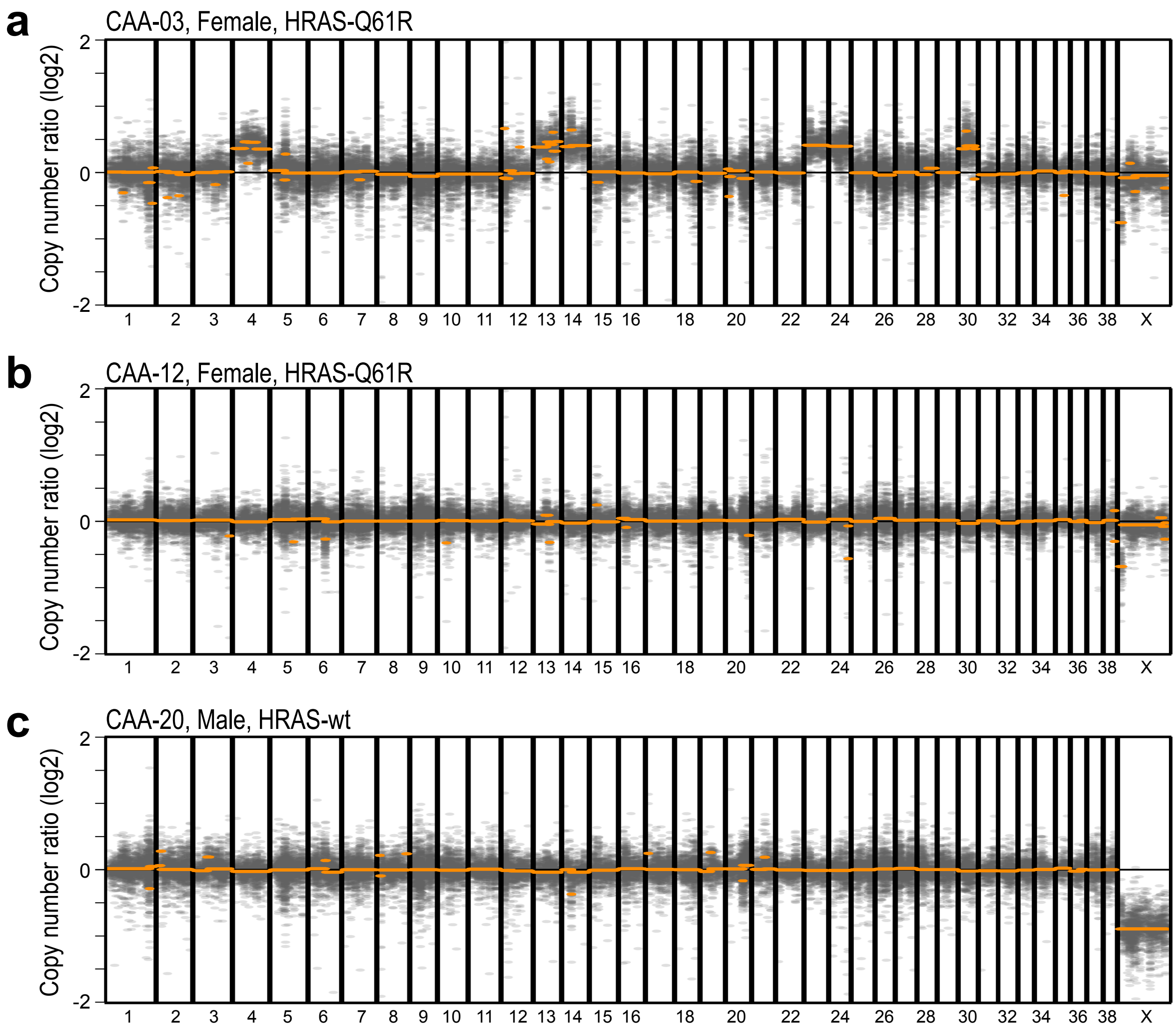

Supplement: Supplementary file 1 — Supplementary Figures [file 41389_2019_119_MOESM1_ESM.pdf]
